# Long-range chromosomal interactions increase and mark repressed gene expression during adipogenesis

**DOI:** 10.1080/15592294.2022.2088145

**Published:** 2022-06-23

**Authors:** Kristina M. Garske, Caroline Comenho, David Z. Pan, Marcus Alvarez, Karen Mohlke, Markku Laakso, Kirsi H. Pietiläinen, Päivi Pajukanta

**Affiliations:** a Department of Human Genetics, David Geffen School of Medicine at UCLA, Los Angeles, CA, USA; b Bioinformatics Interdepartmental Program, UCLA, Los Angeles, CA, USA; c Department of Genetics, University of North Carolina, Chapel Hill, NC, USA; d Internal Medicine, Institute of Clinical Medicine, University of Eastern Finland and Kuopio University Hospital, Kuopio, Finland; e Obesity Research Unit, Research Program for Clinical and Molecular Metabolism, Faculty of Medicine, University of Helsinki, Helsinki, Finland; f Obesity Center, Abdominal Center, Helsinki University Hospital and University of Helsinki, Helsinki, Finland; g Institute for Precision Heath, David Geffen School of Medicine at UCLA, Los Angeles, CA, USA

**Keywords:** Adipogenesis, obesity, chromosomal interactions

## Abstract

Obesity perturbs central functions of human adipose tissue, centred on differentiation of preadipocytes to adipocytes, i.e., adipogenesis. The large environmental component of obesity makes it important to elucidate epigenetic regulatory factors impacting adipogenesis. Promoter Capture Hi-C (pCHi-C) has been used to identify chromosomal interactions between promoters and associated regulatory elements. However, long range interactions (LRIs) greater than 1 Mb are often filtered out of pCHi-C datasets, due to technical challenges and their low prevalence. To elucidate the unknown role of LRIs in adipogenesis, we investigated preadipocyte differentiation to adipocytes using pCHi-C and bulk and single nucleus RNA-seq data. We first show that LRIs are reproducible between biological replicates, and they increase >2-fold in frequency across adipogenesis. We further demonstrate that genomic loci containing LRIs are more epigenetically repressed than regions without LRIs, corresponding to lower gene expression in the LRI regions. Accordingly, as preadipocytes differentiate into adipocytes, LRI regions are more likely to contain repressed preadipocyte marker genes; whereas these same LRI regions are depleted of actively expressed adipocyte marker genes. Finally, we show that LRIs can be used to restrict multiple testing of the long-range *cis-*eQTL analysis to identify variants that regulate genes via LRIs. We exemplify this by identifying a putative long range *cis* regulatory mechanism at the *LYPLAL1*/*TGFB2* obesity locus. In summary, we identify LRIs that mark repressed regions of the genome, and these interactions increase across adipogenesis, pinpointing developmental regions that need to be repressed in a cell-type specific way for adipogenesis to proceed.

## Introduction

Forty percent of U.S. adults have obesity, which predisposes them to multiple comorbidities, such as hypertension, type 2 diabetes, non-alcoholic fatty liver, and coronary heart disease [1–4]. In individuals with obesity, adipose tissue can become hypoxic, inflamed, and insulin resistant [5]. Adipocyte hypertrophy is associated with these detrimental outcomes, and is characterized by already-existing adipocytes becoming larger as a result of storing excess fat. An alternative healthier mechanism is hyperplasia, in which preadipocytes differentiate to create new adipocytes to store the excess fat [6]. Hypertrophy is suggested to occur if 1) preadipocytes are programmed to never differentiate; or 2) preadipocytes begin to differentiate, but differentiation pathways are dysfunctional [7]. Clearly, understanding the molecular changes occurring in preadipocytes as they differentiate into adipocytes is important to identify how the regulation of this process may be disrupted in obesity individuals with adipocyte hypertrophy.

Adipocyte differentiation is orchestrated by epigenomic changes at various levels of gene regulation, which in part can be identified by chromatin remodelling that occurs through, for example, promoter and enhancer interactions [8], caused by the three-dimensional chromosomal arrangement in the nucleus [9]. A recent high-throughput method developed to detect these regulatory interactions is promoter Capture Hi-C (pCHi-C). An extension of Hi-C, this method enriches the genome-wide interaction data for promoter interactions, which are enriched for genomic elements involved in regulating gene expression [10]. While there has been an increase in pCHi-C studies in recent years, most of the research has been focused chromosomal interactions that span less than 1–2 Mb [11–13]. This is likely due to the fact that the vast majority of the called interactions fall within shorter interaction distances. One previous study that focused on extremely long-range interactions (ELRIs) greater than 3 Mb in mouse embryonic stem cell (mESC) development found that these interactions are important in developmental priming from pluripotency towards a specified lineage [14]. However, there is a general lack of characterization of LRIs greater than 1 Mb, which is often used as the distance cut-off in studies of *cis* regulation of gene expression, such as *cis*-expression quantitative trait locus (eQTL) analyses.

We observed that the overall proportion of LRIs between 1 and 2 Mb in distance increases more than 2-fold within the first 24 hours of adipogenesis and associates with repressed regional gene expression, suggesting that LRIs might have a biological function in adipocyte differentiation. Our aim was to understand how LRIs and their associated genomic loci differ from short-range interactions (SRIs) through a systematic integration of pCHi-C across three adipogenesis time points (preadipocytes, differentiating preadipocytes (day 1 of differentiation), and differentiated adipocytes (day 14 of differentiation)) with RNA-seq data available from the same time points. We report that the LRIs regulate genes in a repressive manner only once differentiation of adipocytes has been completed, suggesting a cell-type-specific, developmental function of LRIs that can be detected through significant pCHi-C interactions and further assessed for regulatory roles in adipogenesis and gene regulation in other cell types.

## Results

### Long-range interactions are reproducible and increase across adipogenesis

To link regulatory elements involved in adipogenesis to their target genes, we performed promoter Capture Hi-C (pCHi-C) in human primary preadipocytes (PAd), differentiating preadipocytes (day 1, Diff), and fully *in vitro* differentiated adipocytes (day 14, Adip) (Supplementary Table 1; see Methods). We identified 84,580, 97,116, and 109,831 intrachromosomal interactions in the PAd, Diff, and Adip time points, respectively (Supplementary Table 2). Previous pCHi-C publications have generally filtered out long-range interactions (LRIs), but there are inconsistencies surrounding which distance should be regarded as the upper cut-off. For example, studies have filtered out interactions greater than 1 Mb [15] or 2 Mb [13], while others specifically examine extremely long-range interactions (ELRIs) greater than 3–10 Mb [14,16]. To determine which interaction distances are reliable for the pCHi-C data across adipogenesis at the sequencing depth in this study, we first assessed the reproducibility of the interactions between biological replicates.

**Figure 1.**
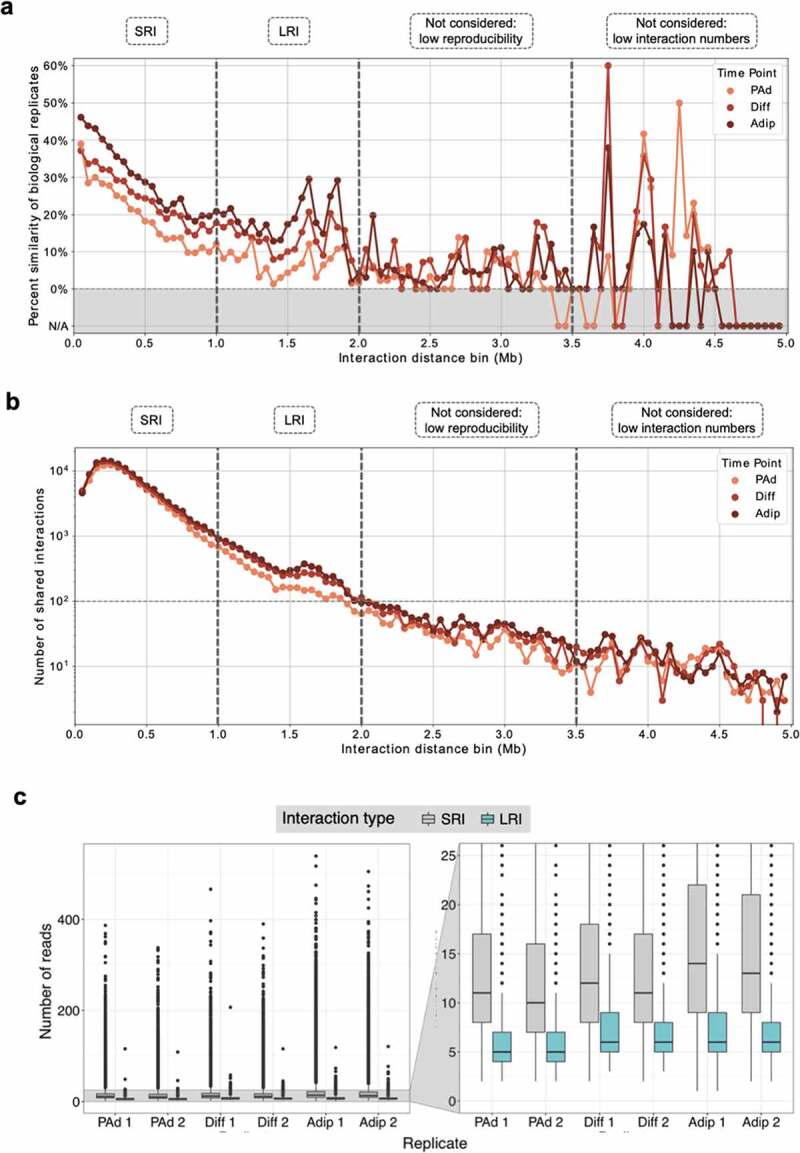
**Long-range interactions are reproducible and increase across adipogenesis**. (a) Percentage of interactions in 50 kb bins that are shared between biological replicates for the PAd, Diff, and Adip timepoints. N/A shaded regions indicate bins that contained fewer than 10 interactions. Vertical dashed lines indicate the different interaction categories that were included (SRIs, LRIs) or excluded (interactions >2 Mb) in downstream analyses. (b) Number of interactions that are shared between both biological replicates within the 50 kb bins for the PAd, Dif, and Adip timepoints. Vertical dashed lines indicate the different interaction categories that were included (SRIs, LRIs) or excluded (interactions >2 Mb) in downstream analyses. (c) Distribution of sequencing reads supporting significant SRI (<1 Mb, grey) or LRI (≥1 Mb, teal) in each biological replicate. PAd indicates preadipocytes; Diff, differentiating PAd; Adip, adipocytes; Mb, megabases; SRI, short-range interaction; and LRI, long-range interaction.

We called significant interactions using CHiCAGO [17] on each pCHi-C library (n = 6, two biological replicates per adipogenesis stage) separately. Given that significant interactions can be variable based on the caller used, we verified interactions, especially LRIs, using the Capture Hi-C ANalysis Engine (CHiCANE) tool [18] (Supplementary Figure 1). We found that >97% of CHiCAGO LRIs were also called by CHiCANE at all three adipogenesis time points. We then stratified the interactions into 50 kb distance bins and calculated the proportion of the total number of interactions within a given bin that is seen in both biological replicates of a given adipogenesis stage (for the full quantitative analysis, see Supplementary Tables 3–5). We found that the reproducibility between biological replicates at the same adipogenesis stage is highest at shorter interaction distances (50–100 kb), and decreases with increasing distance (Figure 1a). While the drop in reproducibility at longer distances is consistent with the reduction of interaction contact frequencies at these distances [17], we were surprised to see that there were peaks indicating substantially increased concordance between pCHi-C biological replicates occurring at longer interaction distances. This was especially the case for interaction distances of 1.5–2 Mb, where the percent reproducibility between biological replicates reached similar values to interactions spanning ~500 kb (Figure 1a). For interactions spanning 2–3.5 Mb, the replicate concordance often approaches 0% (Figure 1a), which suggests that these interactions may not be reliable. For interactions spanning 3.5–5 Mb, while there seems to be a strong peak in biological replicate concordance, there are much fewer interactions (Figure 1b), making the extent of the concordance between biological replicates difficult to interpret. Given these distance-based observations, we chose to limit our downstream analyses to interaction distances less than 2 Mb. It is important to note that these LRIs are supported by a reasonable number of sequencing reads (median of 6 reads, 4–8 IQR), albeit fewer reads than interactions spanning less than 1 Mb (median of 12 reads, 8–19 IQR) (Figure 1c). Additionally, interactions are distributed across the three time points (Supplementary Figure 2). Over 40% of all LRIs are shared by either two or all three time points, indicating that they are likely not an artefact emerging from solely one dataset. Taken together, the reproducibility between biological replicates and sufficient sequencing coverage of the LRIs across adipogenesis support that these LRIs are not false positives in the interaction calling.

When first characterizing the interaction dynamics occurring in adipogenesis, we realized that the overall number of interactions increases during differentiation, as does the number of interactions unique to each time point (Supplementary Table 2). This is in line with the re-wiring of chromosomal interactions through the differentiation of preadipocytes into fully differentiated adipocytes, as reported previously [11]. Looking closer at the data, we realized that the relative proportion of LRIs increases 2.4-fold (from 1.1% in PAd to 2.6% in Diff) in the first 24 hours of adipogenesis (Supplementary Table 2). This marked increase in the percent of LRIs suggests that they may be an important genomic regulatory mechanism during adipogenesis, potentially behaving in a manner that is distinct from short-range interactions (SRIs, <1 Mb distance). In this paper, we sought to characterize the regulatory potential of these LRIs and determine whether there are functional differences between SRIs and LRIs.

### *LRIs provide putative mechanisms for mediating* cis *gene regulation at distances greater than 1 Mb*

**Figure 2.**
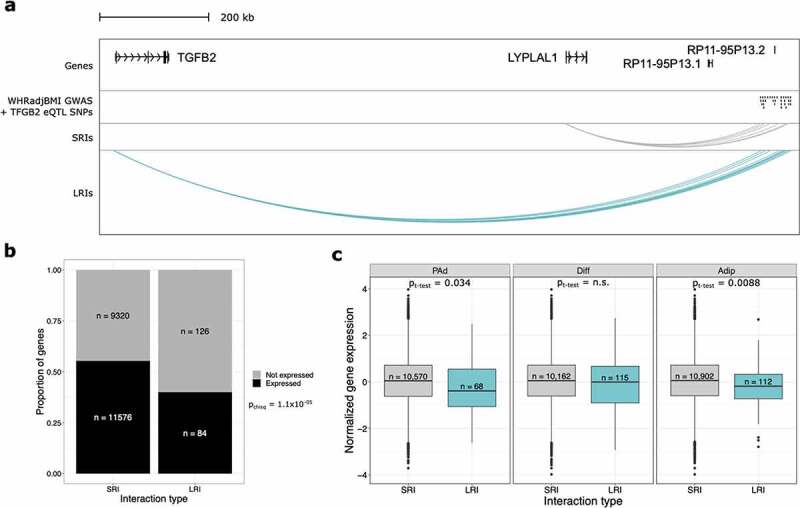
Long-range interactions are involved in gene regulation and associated with repressed gene expression. (a) WashU Genome Browser snapshot of one WHRadjBMI GWAS signal at the *TGFB2/LYPLAL1* locus show that both genes are involved in physical interactions identified through pCHi-C. Only *TGFB2* is significantly regulated by the GWAS SNPs and involved in interactions with the SNPs. The long non-coding RNAs, *RP11-95P13.1* and *RP11-95P13.2* are regulated by the WHRadjBMI GWAS SNPs but were not baited in the pCHi-C design. (b) Bar plot shows the proportion of genes expressed, stratified by whether they are in SRIs or LRIs, excluding genes involved in both SRIs and LRIs. Genes in LRIs are less likely to be expressed. (c) Boxplots show the cell-type-specific expression of genes involved in SRIs or LRIs. Genes in LRIs are more lowly expressed at the Adip time point only, after correcting for multiple testing. Genes in LRIs are nominally more lowly expressed at the PAd time point. WHRadjBMI indicates waist-to-Hip ratio adjusted for body mass index; eQTL, expression quantitative trait locus; SRIs, short-range interactions; LRIs, long-range interactions; PAd, preadipocytes; Diff, differentiating PAd; and Adip, adipocytes.

One mechanism through which genetic variants can regulate distal genes is through interactions that are identified in pCHi-C [19]. To characterize whether LRIs are regulatory in this regard, we performed an expression quantitative trait locus (eQTL) analysis using adipose tissue RNA-seq data from the METabolic Syndrome In Men (METSIM) Finnish population-based cohort [19,20]. We can identify putative mechanisms for long-range *cis*-mediated gene regulation by testing for the effects of SNPs that land within the LRIs on the genes they interact with, while also reducing the multiple testing burden for identification of statistically significant interactions at these distances (see Methods). We identified 17 genes that are involved in pCHi-C LRIs and interact with a SNP that regulates the gene’s expression (Supplementary Table 6). One of these genes, *TGFB2*, is interacting with a GWAS signal for waist-to-hip ratio adjusted for body mass index (WHRadjBMI) [21] (Figure 2a). Notably, these WHRadjBMI GWAS *cis*-eQTL SNPs are also interacting with the *LYPLAL1* gene through SRIs. However, they do not regulate *LYPLAL1* in a conventional *cis-*eQTL analysis that limits SNP-gene distances to 1 Mb or less [19]. The GWAS SNPs do regulate two long non-coding RNAs (lncRNAs) at distances shorter than 1 Mb, but the promoters of these lncRNAs were not baited in the pCHi-C and thus we cannot be sure whether they are regulated via physical interactions at short distances. Thus, extending the SNP-gene distance limit to incorporate LRIs revealed that this GWAS locus is capable of being a *cis* regulator, likely through physical interactions at long distances. This suggests that novel biology can be revealed when considering interactions spanning distances greater 1 Mb, establishing the functional role of LRIs to regulate genes.

### LRIs repress gene expression and mark epigenetically silenced loci

We hypothesized that if LRIs are functionally distinct from SRIs, then there would be differences in expression levels between genes involved in SRIs compared with those involved in LRIs. To test this, we used previously published RNA-seq quantifications of gene expression across adipogenesis from human immortalized adipose tissue derived mesenchymal stem cells (AT-hMSC-TERT4) [22]. This work included RNA-seq at high temporal resolution across adipogenesis, enabling us to integrate these data with our three adipogenesis time points (Supplementary Figure 3; see Methods). We first determined whether genes involved in LRIs have different likelihoods of being expressed than genes involved in SRIs (i.e., passing the expression threshold for the adipogenesis RNA-seq data set in Rauch, *et al* [22].). We found that genes involved in LRIs are less likely to be expressed than genes in SRIs (*p*_chisq_ = 1.1x10^−05^) (Figure 2b). Next, we determined the expression levels of the genes that are involved in LRIs compared with those involved in SRIs. Importantly, we performed this analysis in a cell-type-specific manner using the RNA-seq data from the 1d, 3d, and 14d time points combined with the pCHi-C interactions from the PAd, Diff, and Adip time points, respectively. We found that genes in LRIs exhibit lower expression than genes involved in SRIs, but only in the Adip time point (*p*_t-test_ = 8.8x10^−03^) (Figure 2c). Combined with the lower likelihood of genes in LRIs being expressed, this suggests that LRIs have repressive effects, but these effects are most established after the terminal differentiation of adipocytes (Figure 2b,c).

**Figure 3.**
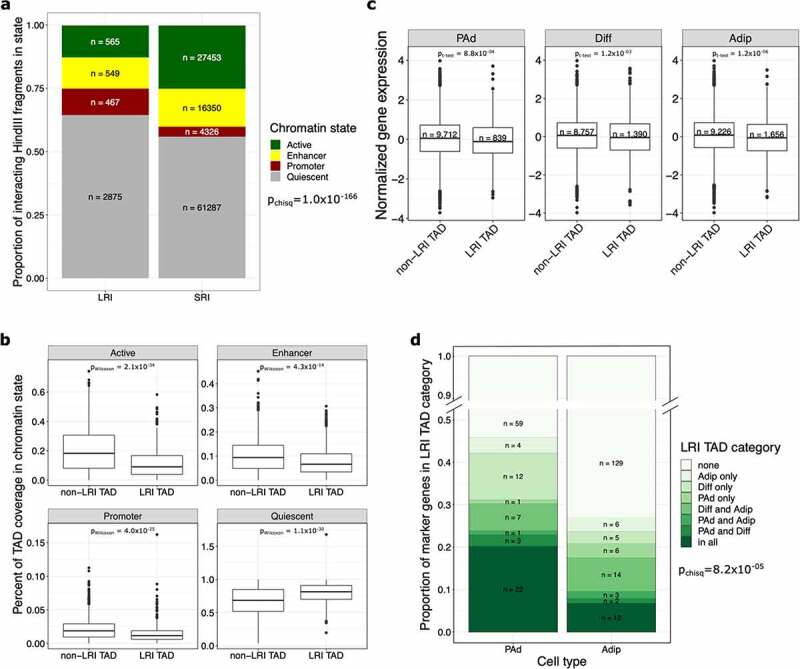
TADs containing LRIs are epigenetically repressed and lack actively expressed cell type marker genes. (a) Bar plot shows the proportion of interacting fragments in the indicated chromHMM chromatin state, stratified by whether the interacting fragment is involved in LRIs or SRIs. Fragments involved in both LRIs and SRIs were removed. LRIs are made up of more quiescent or promoter interacting fragments, whereas SRIs are made up of more actively transcribed or enhancer interacting fragments. (b) Boxplots show the coverage of chromHMM chromatin states in TADs, stratified by whether the TAD lacks LRIs (non-LRI TAD) or has at least one LRI (LRI TAD). LRI TADs have a significantly higher coverage of quiescent chromatin states. (c) Boxplot shows the expression of genes involved in SRIs at the PAd, Diff and Adip time points, stratified by whether the gene lands in an LRI or non-LRI TAD. Genes that land in LRI TADs, even when they are not involved in LRIs themselves, are expressed at lower levels than genes that land in non-LRI TADs. (d) Bar plot shows the proportion of PAd and Adip marker genes that land in the different LRI TAD categories. The y-axis is truncated between 0.5 and 0.9. For the chi-square test, only LRI TAD categories that had at least 5 expected counts for both PAd and Adip marker genes (all, none, Diff only, and Diff and Adip categories) were kept in the analysis. PAd marker genes are more likely to land in the all or Diff only LRI TAD categories, whereas Adip marker genes are more likely to land in non-LRI TADs. LRIs indicates long-range interactions; SRIs, short-range interactions; TAD, topologically associating domain; PAd, preadipocyte; Diff, differentiating preadipocyte; and Adip, adipocyte.

Given that the genes in LRIs have lower expression than genes in SRIs, we next sought to determine whether the promoter-interacting fragments are more epigenetically repressed based on the chromatin state segmentation from chromHMM [23]. We categorized each interacting fragment as active, promoter, enhancer, and quiescent states from the MSC-derived adipocyte cultured cell (MSC-Ad) chromHMM annotations (see Methods). We found that the LRI fragments were more likely to have quiescent or promoter states and less likely to have active or enhancer states when compared with the SRI fragments (*p*_chisq_ = 1.0x10^−166^) (Figure 3a).

We next asked whether the quiescent states within the LRIs reflect the state of the surrounding genomic locus, rather than the interacting fragments alone. To test whether regions containing LRIs have differential chromHMM coverage compared with regions without LRIs, we split the genome into discrete regions using the topologically associating domains (TADs) identified previously in human mesenchymal stem cells (MSCs) [24]. We calculated the coverage of the active, promoter, enhancer, and quiescent states from the MSC-Ad in TADs that contain LRIs (LRI TADs) separately from those that do not contain LRIs (non-LRI TADs) (see Methods). The LRI TADs exhibited a higher coverage of quiescent states (*p*_Wilcoxon_ = 1.1x10^−30^) and the non-LRI TADs exhibited a higher coverage of the measures of active gene regulation: enhancers (*p*_Wilcoxon_ = 4.3x10^−14^), promoters (*p*_Wilcoxon_ = 4.0x10^−25^), and actively transcribed states (*p*_Wilcoxon_ = 2.1x10^−34^) (Figure 3b). Furthermore, we called super-enhancers within the same adipogenic time points (see Methods) and found that the LRI TADs do not significantly overlap with these super-enhancers (p_hypergeom_ = 0.11, 0.19, and 0.08 in PAd, Diff, and Adip, respectively).

Interestingly, we found that genes located within the LRI TADs, but not detected as being involved in LRIs, are still more lowly expressed than genes located outside of the LRI TADs at all adipogenesis time points (*p*_t-test_<1.2x10^−03^) (Figure 3c). This suggests that the lower gene expression observed in the LRI-containing regions is not driven by the genes involved in LRIs alone. Thus, LRIs may mark the overall repressive gene regulation in the locus, rather than only in the LRI interacting fragments. This suggests that LRIs, are a marker – and possible consequence – of the overall repressed state of the surrounding genomic regions. Concordant with this finding, in adipocytes, the LRI TADs are enriched for B compartment regions when compared to non-LRI TADs (Supplementary Figure 4). We also observed that the LRIs in LRI TADs are enriched for motifs of chromatin architecture remodellers and mediators, such as HNF1B, FOXA3, PAX3, CTCF, and HINFP, when compared to the SRIs in LRI TADs (Supplementary Tables 7 and 8), suggesting that LRIs may demarcate epigenetically remodelled loci. Taken together, these results suggest that regions containing LRIs are more epigenetically repressed than the regions of the genome that contain short-range, but not long-range, interactions.

### TADs containing actively expressed cell type marker genes lack LRIs

To determine whether the epigenetic repression we observed in the LRI TADs occurs in a time-point-specific manner, we next categorized the TADs based on whether they contained LRIs in a given adipogenesis time point (Supplementary Figure 5). We found that 177 of the 575 (32.5%) LRI TADs contained LRIs in all 3 time points (Supplementary Figure 5), and 148 (25.7%) LRI TADs contained LRIs in only two time points. Notably, 250 (45.9%) of the LRI TADs only contained LRIs at one time point, altogether indicating that LRIs exhibit dynamic localization across adipogenesis (Supplementary Figure 5). This suggests that LRIs could be functioning to repress gene expression in a cell-type-specific manner. We therefore asked whether cell type marker genes, identified in our previous subcutaneous adipose single nucleus RNA-seq (snRNA-seq) data analysis [25], are distributed differentially across the various time-point-specific categories of LRI TADs. We tested how many of the 113 PAd and 179 Adip marker genes were present in each of the TAD categories (Figure 3d). We found that actively expressed cell type marker genes were more likely to land in TADs that lack LRIs at that adipogenesis time point (*p*_chisq_ = 8.2x10^−05^). First, we observed a higher percentage of PAd marker genes in the shared and Diff-only LRI TADs, in line with these genes needing to be repressed throughout adipogenesis (shared) or transiently repressed at the induction of terminal adipocyte differentiation (Diff-only). Second, we found that there was a higher percentage of Adip marker genes in the non-LRI TADs, consistent with a lower likelihood for these genes being epigenetically repressed through LRIs during the preadipocyte to adipocyte transition. These results highlight a potential role for LRI TADS in the dynamic regulation of cell-type-specific genes throughout the differentiation process.

## Discussion

Promoter Capture Hi-C (pCHi-C) is used to study promoter interactions that regulate gene expression [10,13,15,16]. Given that both biological and technical factors limit the detection of long-range interactions (LRIs), these interactions are often filtered out of pCHi-C datasets and are thus understudied. Here, we investigated whether we could leverage pCHi-C interaction dynamics across three preadipocyte differentiation time points to uncover novel adipogenesis biology, with a focus on the distinction between short- and long-range interaction effects on gene regulation. We systematically assessed the differences between interactions that span distances of less than 1 Mb (short-range interactions, SRIs), or 1–2 Mb (long-range interactions, LRIs). We integrated multiple levels of genetic, epigenetic and transcriptomic data to obtain an organizational and gene-level view on how LRIs function within the genome. We found significant evidence that genes involved in LRIs have lower expression, which is driven by epigenomic silencing across the loci in which the LRIs reside. These LRIs are present and increase across adipogenesis and are thus likely important for regulating key genes for cell-type-specific gene repression. Overall, we discovered that LRIs demarcate repressive epigenomic signatures in a time-point-dependent manner across adipogenesis.

LRIs increase more than two-fold within the first 24 hours of adipogenesis. A large proportion of these LRIs (>30%) land in the same topologically associating domains (TADs) across all adipogenesis time points studied. However, many also land in regions in a time-point-specific manner, highlighting the dynamic nature of the LRIs during adipogenesis. LRI-containing loci may require epigenetic silencing to allow for certain developmental processes to proceed. In line with this, preadipocyte (PAd) marker genes that were identified through adipose single nucleus RNA-seq (snRNA-seq) are more likely to be found in regions that contain LRIs across all adipogenesis time points, or only in the day 1 of differentiation time point (Diff). Combined with our discovery that LRIs mark epigenetically repressed loci and likely repress gene expression, this suggests that LRIs may be markers or signals of the overall repressed state of their surrounding genomic regions. Noteworthy, this would also allow a wider use of pCHi-C data in the identification of cell-type-specific repressed domains, thus suggesting a novel application for the pCHi-C method that is generally thought to be limited in terms of its capacity to infer the higher order structures of the genome aside from promoter-enhancer contacts. In the context of adipogenesis, our results on the repressive LRI loci suggest that genes important in maintaining the adipocyte progenitor cell population are ultimately repressed to enable adipogenesis to proceed. In future studies, comparing the genomic regions in which LRIs land across cell types from different developmental trajectories, such as from different germ layers, may provide insight into novel important regulators of these different lineages. Further, given that LRIs contain motifs for epigenetic remodelling TFs that are often associated with mediating chromatin loop formation, future studies are warranted to distinguish whether LRIs mark repressed loci or help mediate repression of loci through, for example, the creation of new insulating chromatin loops. Additionally, further studying LRIs would shed light on novel *cis* gene regulation, such as those occurring through distal SNP-gene regulatory pairs. Here, by incorporating LRIs into *cis*-eQTL analyses, we expanded our understanding of the *TGFB2/LYPLAL1* GWAS locus for WHRadjBMI. Since conventional *cis*-eQTL analyses that limit the *cis* regions to 0.5 to 1 Mb would miss interactions, such as the one we observed with *TGFB2*, using distal interactions provides a more complete picture of the regulatory interaction landscape that could contribute to variation in human traits.

Our study has several limitations and future directions. First, it is important to note that the repressive effects we observed associating with LRIs are not always predictable. For example, while there is an overall mean reduction in expression levels for genes involved in LRIs compared with those involved in SRIs, some genes involved in LRIs are still expressed at appreciable levels. Thus, our findings support the implementation of future targeted analyses and downstream experimental assessments to improve our understanding of whether LRIs act as repressors or simply mark repressed loci. Second, we recognize that the distance cut-off for pCHi-C LRIs in this study and others can be rather arbitrary or highly dependent on the experimental design and quality of the data, such as the number of replicates used and the sequencing depth. We used a systematic approach to define LRIs based on the reliability of 1–2 Mb interaction distances, as assessed by their biological reproducibility concordance and interaction numbers. However, the extent to which the repressive effects we observed are specific to these distances is not yet clear. Furthermore, it is important to study these LRIs in a greater variety of cell types in order to evaluate their possible universal repressive role.

In conclusion, in this study we investigated the role of LRIs in repressive mechanisms across preadipocyte differentiation. While there is much left to explore regarding the mechanisms through which LRIs act as gene and locus repressors, we provide insight into their differential roles in gene regulation relative to SRIs, and show that these interactions are likely key dynamic genomic regulators in adipogenesis.

## Methods

### Preadipocyte (PAd) cell culture

We grew human primary white PAd (ZenBio, lot L120116E) in PAd growth medium (PromoCell C-27410) supplemented with 1% Gibco Penicillin–Streptomycin (ThermoFisher 15,140,122). The cells were maintained in a 37-degree incubator with 5% CO_2_ during the culturing period. For the PAd pCHi-C and ATAC-seq, cells were grown to <90% confluency in two (pCHi-C) or four (ATAC-seq) biological replicates. For the differentiating PAd pCHi-C and ATAC-seq, PAd were grown to 100% confluency (7–10 M cells). We differentiated the PAd for 24 hours (Diff) using PAd differentiation medium (PromoCell C-27436) in two (pCHi-C) or four (ATAC-seq) biological replicates. For the 14-day time point (Adip), we used our previously published pCHi-C data [19,26] and created ATAC-seq libraries in four biological replicates.

### Promoter capture Hi-C (pCHi-C) library preparation and sequencing

We fixed the nuclei and prepared the pCHi-C libraries in two biological replicates each of the PAd and Diff, as described previously [19,26]. The libraries were sequenced using paired-end sequencing on the Illumina HiSeq 4000, producing an average of 104 M ± 14 M reads.

### pCHi-C computational analysis

To compare the PAd and Diff pCHi-C to the pCHi-C in fully differentiated adipocytes (Adip) [19,26], we down-sampled the previously published Adip pCHi-C libraries to the median of the PAd and Diff read depth (101 M) reads and reprocessed the data with the PAd and Diff data. We processed the sequencing data using the Hi-C User Pipeline (HiCUP) v0.5.9 [27] as described previously [19,26]. The pipeline involves aligning the sequencing reads to the human hg19 reference genome, and then filtering the reads for experimental artefacts that are typical for pCHi-C. After read processing, we detected significant interactions using the Capture Hi-C Analysis of Genome Organization (CHiCAGO) software v1.1.1 [17] using a CHiCAGO score threshold of 5 to define significant interactions. We filtered out inter-chromosomal interactions. To test the concordance in interaction calls between biological replicates, the CHiCAGO pipeline was run on each biological replicate separately. For the final dataset, we called interactions on both biological replicates together in each cell type separately. Based on our biological replicate concordance analyses, we filtered out interactions greater than 2 Mb. We also assessed the reproducibility of the significant interactions called by CHiCAGO by comparing to an alternative capture Hi-C interaction caller, Capture Hi-C ANalysis Engine (CHiCANE) [18]. Starting from the processed reads, we merged replicates using the ‘weighted-sum’ option, and specified the Poisson distribution for modelling the expected read counts for the interactions. Multiple testing correction was done at the global level, rather than at the bait level.

### Genomic distribution of long-range interactions (LRIs)

The distribution of the number of LRIs across the 22 autosomal chromosomes, normalized to the length of chromosome 22 indicated that chromosome 6 contains a larger number of long-range interactions compared with all of the other chromosomes. These interactions were located in a cluster of Histone (*HIST*) genes on chromosome 6, which contains 493 unique long-range interactions across the three adipogenesis stages. Importantly, removing interactions in the *HIST* locus did not drastically affect the high biological replicate concordance at interactions distances greater than 1 Mb. We therefore removed this locus from all analyses to avoid the outlier effects.

### Integrating RNA-seq data across adipogenesis

We integrated our primary PAd, Diff, and Adip pCHi-C data with RNA-seq data from a previously published study of adipogenesis at high temporal resolution [22]. This study used human immortalized adipose tissue derived mesenchymal stem cells (AT-hMSC-TERT4). To determine which time points from Rauch *et al*. correspond most closely with the primary cells we are using in this work, we used RNA-seq data produced at PAd and Diff as a part of a Finnish monozygotic twin study [28]. We took the top 5000 expressed genes at each time point in the adipogenesis study and measured their correlation with the human primary PAd and Diff RNA-seq data. We found that the PAd corresponds most closely with the gene expression measures at 1d in the adipogenesis study, and the Diff correlation maximizes and plateaus at 3d in the adipogenesis study. We therefore used the 1d RNA-seq data to compare with our PAd pCHi-C data; 3d RNA-seq to compare with Diff pCHi-C; and the 14d RNA-seq to compare with the Adip pCHi-C data.

### Defining LRI and non-LRI TADs

We downloaded the TAD locations called in human mesenchymal stem cells (MSCs) [24]. TADs have been shown to be largely conserved across cell types [24] and MSCs are a precursor cell type to preadipocytes. In line with this, we found that only 5.4% of the PAd pCHi-C interactions crossed the MSC TAD boundaries, suggesting that these boundaries provide reasonable demarcation of PAd TAD boundaries. We determined which TADs have at least one end of a long-range interaction (>1 Mb) within the TAD in each of the time points (PAd, Diff, and Adip) separately, and called these LRI TADs. TADs that do not contain any end of an LRI, but contain at least one end of a pCHi-C interactions, were called non-LRI TADs. TADs that do not contain any pCHi-C interactions were excluded from our analyses.

### ChromHMM chromatin state assignment to interacting fragments and calculation of TAD coverage

We downloaded the chromHMM [23] 25-state segmentation from the Roadmap Epigenomics Project for the MSC-derived adipocyte cultured cells. We determined the TAD coverage for each subset of chromHMM states (enhancers, promoters, quiescent, and active) using the bedtools [29] intersect function and dividing by the length of the interacting *Hin*dIII fragment or TAD for the fragment state assignment and TAD chromatin state coverage analyses, respectively. For assigning a state to the interacting fragments, we selected the state with the highest coverage relative to all other states in that fragment.

### A/B compartment detection across adipogenesis

To identify active (A) and inactive (B) compartments of the genome, we first performed the omni [30] ATAC-seq [31] protocol as described previously [26], in 4 isogenic biological replicates per time point (PAd, Diff, and Adip). Briefly, we aligned reads to the human reference genome (GRCh37/hg19) using Bowtie2 v2.2.947 (with parameters -k 4 -X 2000 – local), filtering out unpaired mapped reads and reads with MAPQ < 30 (Samtools [32]) and duplicates (marked with Picard Tools). Only reads from the autosomes were retained for downstream analyses. Peaks were called using MACS2 [33] v2.2.7.1 on individual samples to assess the quality control metric, fraction of reads in peaks (FRiP).

We performed the A/B compartment detection as described previously [34]. Briefly, first we binned the ATAC-seq sequencing reads into 100-kb bins across the genome, except for reads landing in blacklisted regions [35]. We calculated the bins per million mapped reads (BPMs) and corrected the log_2_-transformed BPMs for FRiP. Next, we obtained the Spearman’s rank correlation matrix of the bins to get the pairwise bin co-accessibility measures in PAd, Diff, and Adip time points separately. We calculated the first eigenvector of the correlation matrix, by chromosome, using the nipals function in the mixOmics [36] v6.10.9 R package. Since the sign of the eigenvector is arbitrary, we used the known fact that B compartments are generally more correlated than the A compartments [34]. We thus correlated the eigenvector with the compartment connectivity (sum of the correlation coefficients with all other bins on the chromosome), and ensured that A compartments (positive values in the eigenvector) are negatively correlated with the bin connectivity, changing the sign of the eigenvector if necessary. We smoothed the eigenvector using a simple moving average with a bin size of 3 to obtain the final set of A/B compartments.

### Testing super enhancer overlap with LRI TADs

We downloaded the raw FASTQ ChIP-seq data for the H3K27ac histone mark and MED1 at the day 1 (PAd), day 3 (Diff), and day 14 (Adip) [22] -differentiated cells from bone marrow derived stromal stem cells (BM-hMSC-TERT4) from the GEO database (accession code GSE113253). Sequencing reads were aligned to the hg19 reference genome using Bowtie2 v2.2.9 [37] (with parameters -k 4 – local), filtering out unmapped reads and reads with MAPQ < 30 (Samtools [32]) and duplicates (marked with Picard Tools).

Peaks were called on each biological replicate separately using MACS2 [33] v2.2.7.1 and then consensus peaks were called on both replicates together to run the irreproducible discovery rate (IDR) analysis in order to identify reproducible peaks across both replicates. Only MED1 peaks that overlapped with H3K27ac peaks were retained as the constituent peaks for downstream analyses to identify super-enhancers. The ROSE algorithm [38,39] was used to call super-enhancers based on the MED1 ChIP-seq alignments. We then assessed how many super-enhancers land within the TADs containing LRIs in each of the adipogenesis time points. To test whether the co-occurrence of super-enhancers and LRIs is significant, we shuffled which TADs contain LRIs and re-computed the overlap between the super-enhancers and LRI-containing TADs (n_permutations_ = 10,000).

### Transcription factor motif enrichment in the LRI interacting fragments

We used HOMER v4.9 [40] to investigate the enrichment of known transcription factor (TF) motifs in LRI interacting fragments when compared to SRI interacting fragments within the LRI TADs. We also tested whether the LRI interacting fragments are enriched for TF motifs when compared with all SRI interacting fragments genome-wide, which produced similar results.

### Adipose single-nuclei RNA-seq (snRNA-seq) cell type marker gene identification

PAd and Adip cell type marker genes were identified from adipose snRNA-seq data obtained from frozen adipose tissue (n = 6), as described previously [25]. We selected the unique marker genes for PAd (n = 113) and Adip (n = 179) and overlaid them with the TADs, stratifying the TADs based on whether they exhibited LRIs in a given adipogenesis time point.

### METabolic syndrome in men (METSIM) cohort

The participants in the METabolic Syndrome In Men (METSIM) cohort (n = 10,197) are Finnish males recruited at the University of Eastern Finland and Kuopio University Hospital, Kuopio, Finland, as described previously [19,20,41,42]. The study was approved by the local ethics committee and all participants gave written informed consent. The median age of the METSIM participants is 57 years (range: 45–74 years). The METSIM participants were genotyped using the OmniExpress (Illumina) genotyping array and phased and imputed using SHAPEIT2 v2.17 [43] and IMPUTE2 v2.3.2 [44], respectively. A random subset of the METSIM men underwent an abdominal subcutaneous adipose needle biopsy, with 335 unrelated individuals (IBD < 0.2) using a genetic relationship matrix calculated in PLINK v1.9 [45,46] analysed here using RNA-seq [19,42].

### Long range *cis*-eQTL analysis in the METSIM cohort

We performed *cis*-eQTL analyses in the METSIM cohort using the subcutaneous adipose RNA-seq data (n = 335). We filtered the subcutaneous adipose RNA-seq expression data (FPKMs) to genes expressed (FPKM > 0) in greater than 90% of individuals and employed PEER factor [47] analysis to remove hidden confounders. We conducted PEER factor optimization on chromosome 20 to maximize power for discovery for eQTLs, while ensuring hidden confounders were removed, and thus ended up correcting the METSIM expression data for 22 PEER factors, as described previously [19,42]. The METSIM genotype data was produced using the Illumina HumanOmniExpress BeadChip, Imputed SNP data were filtered using the quality control inclusion criteria of info ≥ 0.8, MAF ≥ 5%, and Hardy–Weinberg equilibrium (HWE) p < 0.00001^19^ [42]. Three genetic principal components (PCs) were included to account for possible population substructure. The *cis*-eQTL analysis was performed using Matrix-eQTL [48] with *cis*-eQTLs classified as those less than 1Mb from either end of a gene and long-range *cis*-eQTLs classified as those over 1 Mb but less than 2Mb from either end of a gene.

### Overlap of long range *cis*-eQTL SNPs and chromosomal interactions

To investigate functional long-range *cis*-eQTL SNPs, we overlapped the imputed *cis*-eQTL SNPs and their target genes with pCHi-C interactions by first overlapping the position of the other end of the looping interaction with the location of the long-range *cis*-eQTL SNP. Simultaneously, we examined the identity of the predicted target gene for the *cis*-eQTL SNP and the gene involved in the looping interaction for a match. Only when both these criteria were fulfilled, was the *cis*-eQTL SNP defined as a long-range looping *cis*-eQTL SNP. All identified long-range *cis*-eQTL SNP-gene pairs were corrected for multiple testing employing the Bonferroni correction, using the number of independent signals (LD R^2^ < 0.2) and a significance threshold of *p*_adj_<0.05. All long-range *cis*-eQTL SNPs were interrogated for whether they are also short-range interacting *cis-*eQTL SNPs, using the following procedure: long-range *cis*-eQTL SNPs that also land in short-range interactions were checked for whether they significantly (FDR < 0.05) regulate the gene whose promoter the eQTL SNP is interacting with in the traditional genome-wide *cis*-eQTL analysis (1 Mb from the gene).

## Supplementary Material

Supplemental Material

## Data Availability

The PAd and Diff pCHi-C data and PAd, Diff and Adip ATAC-seq data are available at GEO under accession number GSE129574. The Adip pCHi-C data are available at GEO under accession number GSE129574.
