# Supplementary material for: Long-range chromosomal interactions increase and mark repressed gene expression during adipogenesis
Source: Epigenetics. 2022 Jun 23;17(13):1849–62. doi: 10.1080/15592294.2022.2088145 (PMC9665133; doi:10.1080/15592294.2022.2088145)
Supplement: Supplemental Material [file KEPI_A_2088145_SM0051.zip › supplementary/SI_resub.docx]

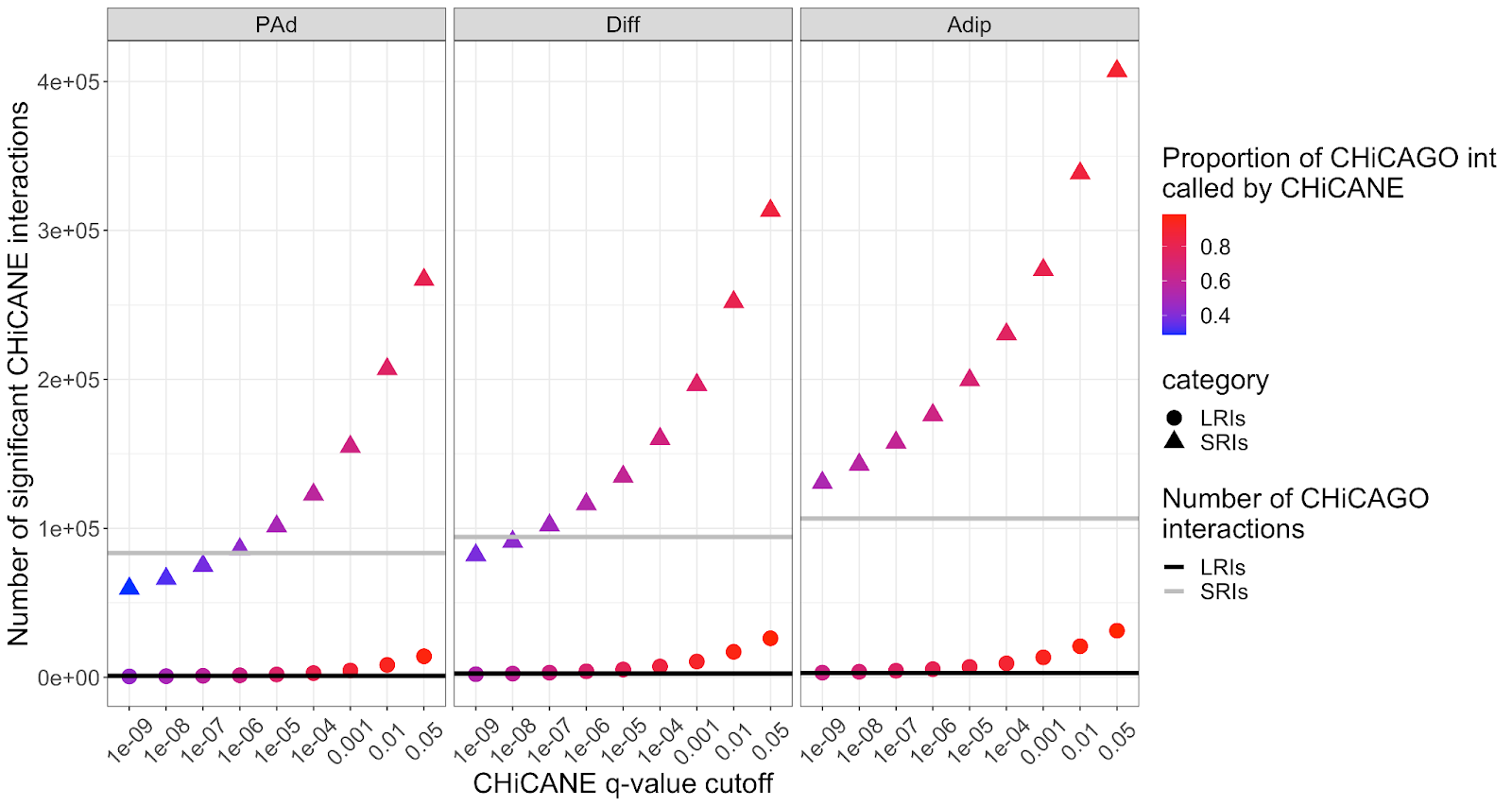
**Supplemental Information**

**Supplementary Figure 1. CHiCAGO interactions are reproducibly detected by the CHiCANE capture Hi-C interaction caller.** Dot plot colors show the proportion of significant CHiCAGO PAd, Diff, and Adip pCHi-C interactions that were also called by the CHiCANE interaction caller, split into SRIs (triangles) and LRIs (circles). The number of significant interactions called by CHiCANE (y-axis) at a q-value cutoff of 0.05 greatly outweighs the number of significant interactions called by CHiCAGO (indicated by the black (LRIs) and gray (SRIs) horizontal lines. Even when stricter q-value thresholds are used for CHiCANE, the reproducibility of the LRIs remains high. PAd indicates preadipocytes; Diff, differentiating PAd; Adip, adipocytes; SRIs, short-range interactions; LRIs, long-range interactions.

**Supplementary Figure 2. Number of cell-type-specific and shared LRIs across adipogenesis.** Venn diagram displays number of LRIs in each adipogenesis time point. LRIs indicate long range interactions; TADS, topologically associating domains; PAd, preadipocytes; Diff, differentiating PAd; and Adip, adipocytes.


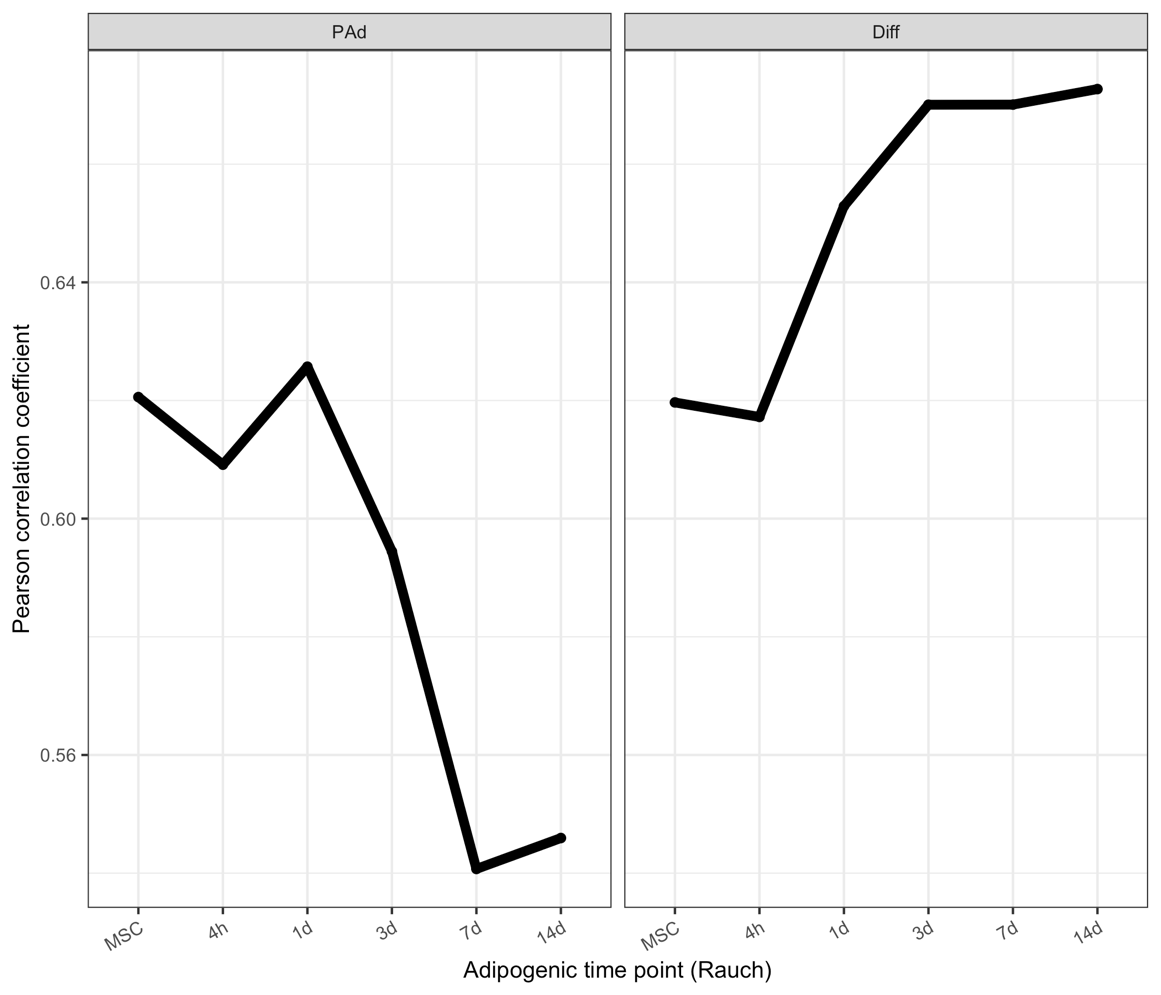


**Supplementary Figure 3. Comparison of primary preadipocyte and differentiating preadipocyte RNA-seq data with AT-hMSC-TERT4 adipogenesis time points**. To compare our primary cell PAd, Diff, and Adip pCHi-C data with RNA-seq data at similar time points in adipogenesis, we used the RNA-seq data produced in human immortalized adipose tissue derived mesenchymal stem cells (AT-hMSC-TERT4) across 6 time points in adipogenesis^1^. As described in the Methods, we first determined which time points across these immortalized cell gene expression trajectories correspond to primary cell PAd and Diff, using RNA-seq data produced in an independent Finnish monozygotic twin study^2^ at these two time points. Using the top 5000 expressed genes at each time point in the adipogenesis study, we measured their correlation with the human primary PAd and Diff RNA-seq data. We found that the PAd corresponds most closely with the gene expression measures at 1d in the adipogenesis study, and the Diff correlation maximizes and plateaus at 3d in the adipogenesis study. We therefore used the 1d RNA-seq data to compare with our PAd pCHi-C data; 3d RNA-seq to compare with Diff pCHi-C; and the 14d RNA-seq to compare with the Adip pCHi-C data. PAd indicates preadipocytes; Diff, differentiating PAd.


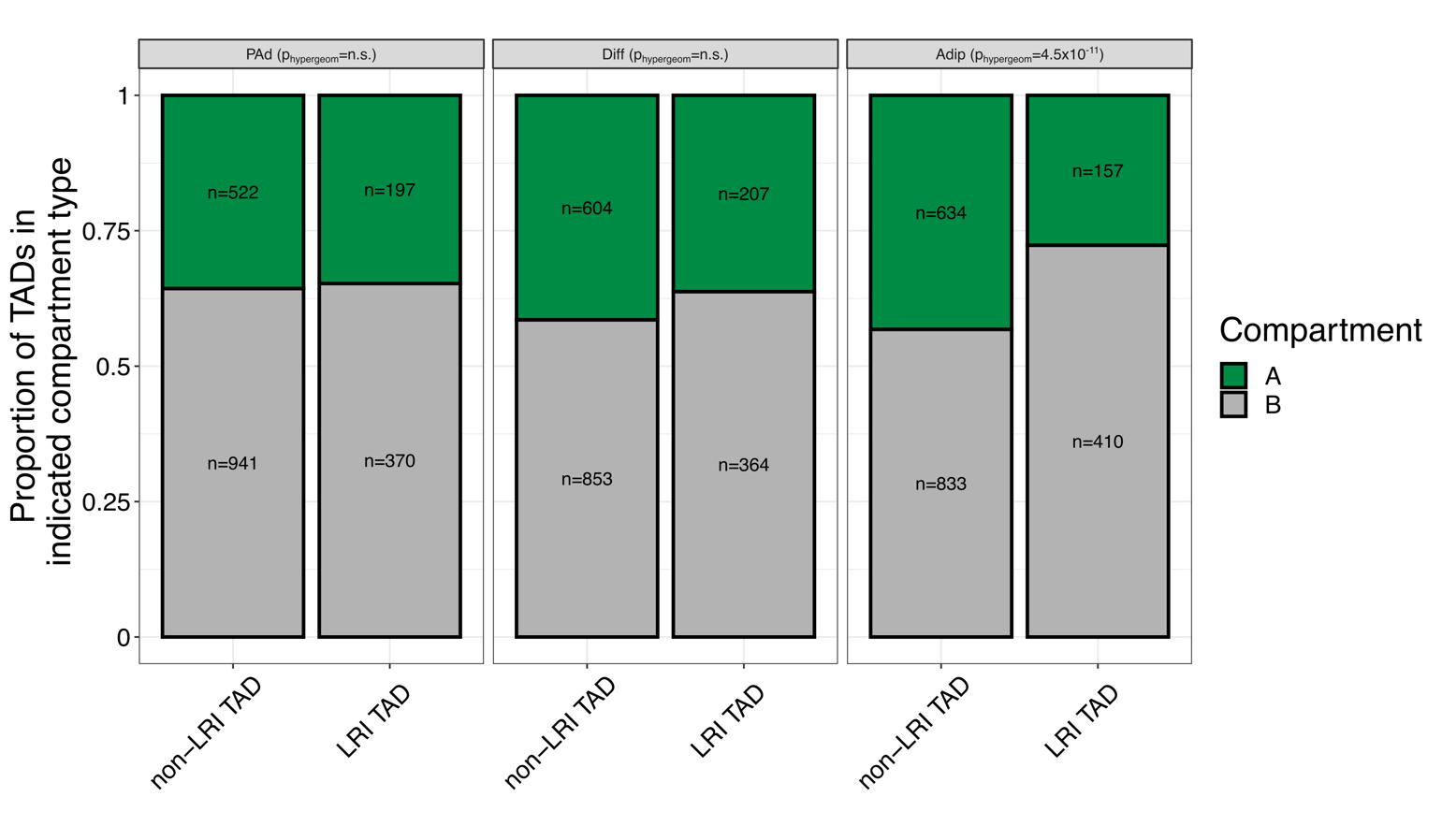


**Supplementary Figure 4. LRI TADs are enriched for B compartments in adipocytes.** Quantification of the proportion of TADs landing A vs. B compartments, stratified by whether the TAD contains LRIs at a given cell type. LRI TADs are significantly enriched in B compartments when compared to non-LRI TADs, only at the Adip time point (p_hypergeom_=4.5x10^-11^).


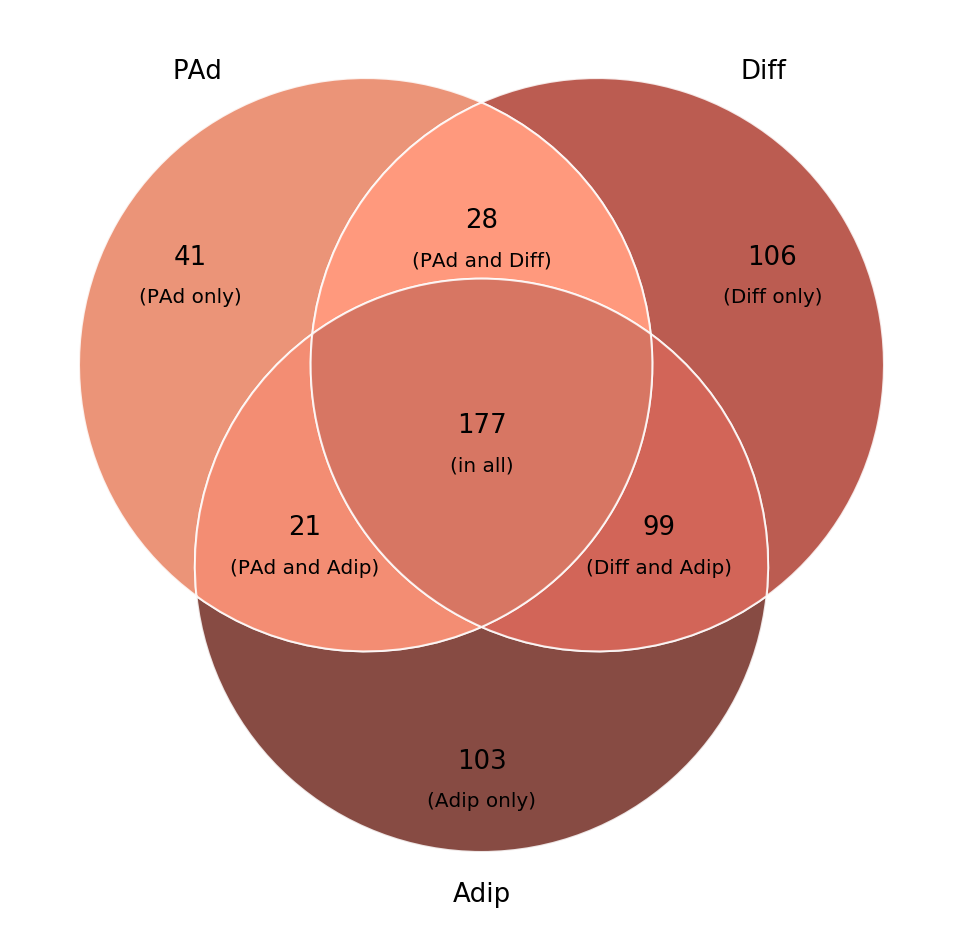


**Supplementary Figure 5. Number of TADs containing LRIs in each adipogenesis time point.** Venn diagram displays the number of TADs that contain LRIs across adipogenesis time points. TADs with no LRIs present in any adipogenesis time point (1,489 TADs) are not shown. LRIs indicate long range interactions; TADS, topologically associating domains; PAd, preadipocytes; Diff, differentiating PAd; and Adip, adipocytes.

**Supplementary Table 1. Promoter Capture Hi-C read processing and capture efficiency.**

| **Library** | **Reads** | **Processed reads*** | **On-target reads** | **Capture efficiency** |
| --- | --- | --- | --- | --- |
| PAd1 | 106,651,878 | 46,453,815 | 37,763,852 | 81% |
| PAd2 | 91,792,535 | 39,354,816 | 32,404,037 | 82% |
| Diff1 | 123,183,077 | 52,589,418 | 41,414,667 | 79% |
| Diff2 | 95,118,778 | 43,957,152 | 36,805,830 | 84% |
| Adip1 | 101,236,761 | 55,920,905 | 48,401,604 | 87% |
| Adip2 | 101,236,761 | 49,574,212 | 43,499,706 | 88% |

*Reads were processed using the Hi-C User Pipeline (HiCUP)^3^, and the total processed reads reflect uniquely aligned, paired, deduplicated reads that were filtered for experimental artifacts related to pCHi-C (e.g., re-ligation).

**Supplementary Table 2. Quantitative report of the investigated promoter Capture Hi-C interaction types.**

| **Interaction spanning distance** | **Interaction type** | **Time Point** | | |
| --- | --- | --- | --- | --- |
|  |  | **PAd** | **Diff** | **Adip** |
| **Intrachromosomal (*cis*) interactions** | **All** | 84,580 | 97,116 | 109,831 |
|  | **Unique (% of total *cis*)** | 25,932  (30.7%) | 28,287  (29.1%) | 42,415  (38.6%) |
|  | **Shared** | 58,648 | 68,829 | 67,416 |
| **Short-range**  **(< 1 Mb) *cis* interactions** | **All (% of total *cis*)** | 83,355  (98.6%) | 94,220  (97.0%) | 106,594  (97.1%) |
|  | **Unique (% of total short-range)** | 25,476  (30.6%) | 26,820  (28.5%) | 40,616  (38.1%) |
|  | **Shared** | 57,879 | 67,400 | 65,978 |
| **Long-range (>= 1 Mb; < 2 Mb) *cis* interactions** | **All (% of total *cis*)** | 968  (1.14%) | 2,511  (2.58%) | 2,842  (2.59%) |
|  | **Unique (% of total long-range)** | 323  (33.4%) | 1,247  (49.7%) | 1,570  (55.2%) |
|  | **Shared** | 645 | 1,264 | 1,272 |

PAd indicates preadipocytes; Diff, differentiating PAd; Adip, adipocytes; and Mb, megabases.

**Supplementary Tables 3-5 (Excel file). Number of reproducible interactions within 50-kb bins across adipogenesis.** Numeric representation of the data presented in the plot in Figure 1a for PAd (Supplementary Table 3), Diff (Supplementary Table 4) and Adip (Supplementary Table 5). Dist column indicates distance bin.

**Supplementary Table 6. Long-range *cis­*-eQTL SNPs that land in LRIs and regulate the interacting gene.**

| **Ensembl ID** | **Gene symbol** | **eQTL SNP** | **Chr** | **Pos** | **Effect allele** | **Interaction distance** | **eQTL effect size** | **eQTL**  **p-value** |
| --- | --- | --- | --- | --- | --- | --- | --- | --- |
| ENSG00000162631 | *NTNG1* | rs77300800 | 1 | 106575719 | A | 1,107,738 | 0.847 | 3.109E-05 |
| ENSG00000092969 | *TGFB2* | rs2820444 | 1 | 219741820 | A | 1,221,796 | -0.369 | 2.068E-05 |
| ENSG00000092969 | *TGFB2* | rs2820442 | 1 | 219754257 | T | 1,236,363 | 0.402 | 5.834E-04 |
| ENSG00000121989 | *ACVR2A* | rs201146059 | 2 | 147481148 | A | 1,116,157 | 0.187 | 4.906E-04 |
| ENSG00000177707 | *PVRL3* | rs6773071 | 3 | 109454187 | G | 1,334,768 | 0.420 | 3.228E-04 |
| ENSG00000068028 | *RASSF1* | rs6771491 | 3 | 51705962 | G | 1,324,089 | -0.304 | 9.343E-04 |
| ENSG00000152784 | *PRDM8* | rs55887993 | 4 | 79889952 | T | 1,214,619 | 0.586 | 2.591E-05 |
| ENSG00000171522 | *PTGER4* | rs148138152 | 5 | 39611089 | CCTT | 1,069,218 | 0.253 | 1.434E-04 |
| ENSG00000155542 | *SETD9* | rs3053501 | 5 | 57757891 | AGG | 1,555,599 | 0.287 | 1.167E-04 |
| ENSG00000203813 | *HIST1H3H* | rs34391493 | 6 | 26022648 | G | 1,755,926 | -0.546 | 6.568E-04 |
| ENSG00000054598 | *FOXC1* | rs1038335 | 6 | 2632108 | C | 1,020,458 | 0.241 | 9.521E-04 |
| ENSG00000118407 | *FILIP1* | rs146201151 | 6 | 74487732 | G | 1,719,775 | -0.474 | 8.835E-04 |
| ENSG00000111799 | *COL12A1* | rs4708108 | 6 | 74679386 | T | 1,231,862 | 0.438 | 7.270E-07 |
| ENSG00000146674 | *IGFBP3* | rs7795923 | 7 | 47124173 | T | 1,164,931 | 0.322 | 9.447E-04 |
| ENSG00000146674 | *IGFBP3* | rs55801677 | 7 | 47207195 | C | 1,246,564 | -0.389 | 7.476E-05 |
| ENSG00000134809 | *TIMM10* | rs12363738 | 11 | 58328579 | T | 1,031,087 | -0.456 | 6.753E-06 |
| ENSG00000205078 | *SYCE1L* | rs71394237 | 16 | 75803981 | C | 1,428,318 | -0.410 | 5.135E-05 |
| ENSG00000181350 | *FAM211A* | rs58804619 | 17 | 18090654 | C | 1,743,599 | -0.419 | 2.108E-04 |
| ENSG00000154734 | *ADAMTS1* | rs56085411 | 21 | 30122895 | T | 1,909,332 | 0.550 | 2.322E-04 |

The Gene symbol/Ensembl ID refers to the gene that is interacting with and significantly regulated by (Bonferroni-corrected *p*<0.05) the eQTL SNP. The eQTL SNP effect allele is listed. The Chr column refers to the chromosome on which the eQTL SNP and regulated genes are located. The Pos column refers to the position of the eQTL SNP in the hg19 human genome build. The interaction distance column shows the distance spanned by the interaction that contains the eQTL SNP in one end and the target gene promoter in the other end. The eQTL effect size and *p-*values are listed for the effect allele. eQTL indicates expression quantitative trait locus and SNP, single nucleotide polymorphism.

**Supplementary Table 7. Top 5 known motifs enriched in LRIs in the LRI TADs over SRI interactions in LRI TADs using HOMER**^4^**.**

| Rank | Name | P-value | # Target Sequences with Motif | % of Targets Sequences with Motif | # Background Sequences with Motif | % of Background Sequences with Motif |
| --- | --- | --- | --- | --- | --- | --- |
| 1 | HNF1B | 1x10^-10^ | 1728.0 | 42.77% | 11453.2 | 37.51% |
| 2 | FOXA3 | 1x10^-10^ | 2215.0 | 54.83% | 15109.5 | 49.49% |
| 3 | PAX3 | 1x10^-10^ | 1572.0 | 38.91% | 10318.9 | 33.80% |
| 4 | CTCF | 1x10^-9^ | 648.0 | 16.04% | 3786.4 | 12.40% |
| 5 | HINFP | 1x10^-9^ | 700.0 | 17.33% | 4151.0 | 13.60% |

Enrichment p-values were derived from the hypergeometric enrichment test of proportion of the listed TF motif in the LRI interacting fragments (i.e., Target sequences) compared with the background set of SRI interacting fragments (i.e., Background sequences) within the LRI TADs, adjusted (Benjamini-Hochberg) for the number of known motifs tested (n=364).

**Supplementary Table 8. Top 5 known motifs enriched in the LRIs over SRI interactions genome-wide using HOMER**^4^**.**

| Rank | Name | P-value | # Target Sequences with Motif | % of Targets Sequences with Motif | # Background Sequences with Motif | % of Background Sequences with Motif |
| --- | --- | --- | --- | --- | --- | --- |
| 1 | CTCF | 1x10^-15^ | 715.0 | 16.47% | 13149.0 | 12.15% |
| 2 | ELK4 | 1x10^-14^ | 1852.0 | 42.65% | 39755.9 | 36.74% |
| 3 | PAX3 | 1x10^-14^ | 1689.0 | 38.90% | 35891.8 | 33.17% |
| 4 | HNF1B | 1x10^-13^ | 1859.0 | 42.81% | 40110.1 | 37.07% |
| 5 | EGR1 | 1x10^-13^ | 1780.0 | 40.99% | 38213.4 | 35.32% |

Enrichment p-values were derived from the hypergeometric enrichment test of proportion of the listed TF motif in the LRI interacting fragments (i.e., Target sequences) compared with the background set of all SRI interacting fragments (i.e., Background sequences) genome-wide, adjusted (Benjamini-Hochberg) for the number of known motifs tested (n=364).

**Supplementary references**

1. Rauch, A. *et al.* Osteogenesis depends on commissioning of a network of stem cell transcription factors that act as repressors of adipogenesis. *Nat. Genet.* **51**, 716–727 (2019).

2. van der Kolk, B. W. *et al.* Molecular pathways behind acquired obesity: Adipose tissue and skeletal muscle multiomics in monozygotic twin pairs discordant for BMI. *Cell Reports Med.* **2**, 100226 (2021).

3. Wingett, S. W. *et al.* HiCUP: pipeline for mapping and processing Hi-C data. *F1000Research* **4**, 1310 (2015).

4. Heinz, S. *et al.* Simple combinations of lineage-determining transcription factors prime cis-regulatory elements required for macrophage and B cell identities. *Mol. Cell* **38**, 576–589 (2010).
